# Iron absorption in raw and cooked bananas: a field study using stable isotopes in women

**DOI:** 10.3402/fnr.v59.25976

**Published:** 2015-02-05

**Authors:** Olga P. García, Mara Martínez, Diana Romano, Mariela Camacho, Fabiana F. de Moura, Steve A. Abrams, Harjeet K. Khanna, James L. Dale, Jorge L. Rosado

**Affiliations:** 1 School of Natural Sciences, Universidad Autonoma de Querétaro, Querétaro, México; 2 HarvestPlus, c/o International Food Policy Research Institute, Washington, DC, USA; 3 Children's Nutrition Research Center Department of Pediatrics, Baylor College of Medicine, Houston, USA; 4 Centre for Tropical Crops and Biocommodities, Science and Engineering Faculty, Queensland University of Technology, Brisbane, Australia

**Keywords:** iron absorption, bananas, iron deficiency

## Abstract

**Background:**

Banana is a staple food in many regions with high iron deficiency and may be a potential vehicle for iron fortification. However, iron absorption from bananas is not known.

**Objective:**

The objective of this study was to evaluate total iron absorption from raw and cooked bananas.

**Design:**

Thirty women (34.9±6.6 years) from rural Mexico were randomly assigned to one of two groups each consuming: 1) 480 g/day of raw banana for 6 days, or 2) 500 g/day of cooked banana for 4 days. Iron absorption was measured after extrinsically labeling with 2 mg of ^58^Fe and a reference dose of 6 mg ^57^Fe; analysis was done using ICP-MS.

**Results:**

Iron content in cooked bananas was significantly higher than raw bananas (0.53 mg/100 g bananas vs. 0.33 mg/100 mg bananas, respectively) (*p<*0.001). Percent iron absorption was significantly higher in raw bananas (49.3±21.3%) compared with cooked banana (33.9±16.2%) (*p=*0.035). Total amount of iron absorbed from raw and cooked bananas was similar (0.77±0.33 mg vs. 0.86±0.41 mg, respectively).

**Conclusion:**

Total amount of absorbed iron is similar between cooked and raw bananas. The banana matrix does not affect iron absorption and is therefore a potential effective target for genetic modification for iron biofortification.

Iron deficiency and anemia affect around 24.8% of the world's population (1) and their consequences are an important public health concern (2). Low iron intake, low iron bioavailability, and the presence of infectious diseases are the main causes of iron deficiency.

Strategies to prevent and control iron deficiency include decreasing poverty, improving of health systems and food security, reducing iron losses, improving iron's bioavailability in foods, and increasing iron intake (3). Supplementation, fortification, and biofortification are strategies that have been implemented worldwide to increase iron intake. However, iron deficiency is still highly prevalent suggesting more strategies are needed to decrease iron deficiency. Because plants are the main source of iron for most people in the developing world, the generation of iron-fortified crops should increase iron intake and have a significant impact on human health.

Banana is a staple food in many countries in the world, specifically Latin America, Asia, and Africa, where the prevalence of iron deficiency is high. In Uganda, for example, 49% of women of reproductive age and 73% of children aged <59 months are anemic; in addition, 88% of women have iron deficiency with or without anemia (4). In Mexico, prevalence of iron deficiency in children <5 years of age is approximately 26% (5). Banana consumption per capita in Uganda is very high: 0.48 kg/day for cooked banana and 0.15 kg/day for raw banana (6). Intake varies markedly depending on the region, with the highest banana consumers in the highlands. Banana intake in Mexico is one of the highest in Latin America (16 kg/year) (7). Thus, in countries where large quantities of bananas are consumed and where iron deficiency is a major health concern, bananas are a potential vehicle for iron fortification.

Iron content in bananas is low, approximately 0.4 mg/100 g of fresh weight. There is a strategy of developing modified lines of bananas to increase their iron content; the target is a 3- to 6-fold increase. To evaluate iron absorption from bananas, both cooked and raw bananas will provide very useful information that could help to predict how the banana matrix affects iron absorption and its potential use to improve iron status. No studies have been made estimating the effect cooking has on iron absorption or how much iron is absorbed from bananas. This information will be useful for countries where iron deficiency is a major health concern and where large quantities of bananas are consumed.

The objective of this study was to evaluate iron absorption from raw and cooked banana using stable isotopes in women living in rural México.

## Materials and methods

### Subjects and place of study

Women between ages 18 and 45 from the rural community of Fuentezuelas, Querétaro, were invited to participate in the study. Subjects received oral and written information before signing a letter accepting voluntarily their participation in the study. The Bioethics Committee from the School of Natural Sciences at the Universidad Autónoma de Querétaro (UAQ) approved the study protocol and the procedures followed were in accordance with the Helsinki Declaration of 1975.

Women were included in the study if they consumed bananas frequently (at least 3 times per week). Exclusion criteria for the participants included obesity [body mass index (BMI)>30 kg/m^2^], any chronic disease such as hypertension or diabetes, any gastrointestinal disease, anemia (Hb<12 g/dL), infection (CRP>3 mg/L), chronic intake of medications, if they had consumed micronutrient supplements that included iron for the past 3 months, pregnant or lactating, and if they had iron deficiency (ferritin<12 µg/L).

Sample size was calculated to detect a biological difference in fractional absorption of iron of 0.1, a standard deviation of 0.08, with an alpha error of 0.05, a power of 90%; also a potential loss of subjects of 10% was included. With these assumptions we needed to include 15 women in each group.

### Study design

Thirty women (34.9±6.6 years) who met the inclusion criteria were randomly assigned to one of two groups:

1. 480 g of raw banana per day for 6 consecutive days (*N=*15).
2. 500 g of cooked banana per day for 4 consecutive days (*N=*15).

One day before the study started, the women were asked to attend the health clinic after an overnight fast, their weight and height were measured and a baseline venous blood sample was taken. A food frequency questionnaire was also applied at this time. The next day each group consumed bananas for breakfast and women were asked not to eat anything prior to the study. Also, the participants were not allowed to consume any food and were asked to stay in the community house for 3 h after consuming the bananas. A reference dose was given on two separate days. In both studies, after 14 days of consuming the reference dose, a final blood sample was taken for the analysis of iron absorption.

The amount of raw and cooked bananas was determined according to the daily consumption in countries where banana is a staple food, such as Uganda (approximately 500 g/day of cooked bananas and 480 g/day of raw bananas) (6). The duration of each study varied according to the amount of iron the subject had to consume daily from the bananas and the iron stable isotope (^58^Fe) that was distributed equally every day in proportion to the intrinsic iron content of raw or cooked bananas.

Iron absorption from the meals was measured with ^58^Fe and ^57^Fe isotopes, respectively, as described by Abrams (8). Bananas were extrinsically labeled with 2 mg of ^58^Fe distributed in the days they received the bananas. The isotopes were given to the subjects in vacuum-sealed glass vials, and women had to consume the isotopes half way through the intake of the bananas. The glass vials had to be rinsed three times with water, and the women had to drink the water also. A reference dose of 6.0 mg of ^57^Fe with 25 mg of ascorbic acid as orange juice was given in the morning for 2 consecutive days (3 mg per day) to subjects in both groups (8 mg of total isotope), without any food.

### Banana preparation

In the two absorption studies, Cavendish bananas obtained from the general market in Querétaro, México, on the same day of the studies were used. This type of banana was selected because studies are being made to develop genetically modified lines to increase iron absorption in the same type at the Centre for Tropical Crops and Biocommodities, Queensland University of Technology, Australia.

The cooked banana was prepared based on the Ugandan traditional dish known as *matooke*. Following the traditional recipe, green bananas were peeled, chopped, and then wrapped in banana leaves and steamed for 45 min until soft enough to mash and serve. The cooked bananas were prepared daily in the morning and were served warm, with no sauce or spice added. The raw bananas were served ripe, which was determined by their yellow peel.

### Anthropometry and body composition

Weight, height, and waist and hip circumferences were measured in duplicate by trained personnel following standard procedures (9). For the present study, obesity was considered with a BMI≥30 kg/m^2^, and overweight with a BMI 25–29.9 kg/m^2^ (10).

### Iron and phytate content

Iron and phytate content were analyzed in duplicate samples of raw and cooked bananas used in the studies. Iron content was analyzed by atomic absorption spectrometry (Mod AAnalyst 800, Perkin Elmer, CT, USA) using the corresponding standards. Phytate was analyzed in duplicate by spectrophotometry using the technique by Vaintraub and Lapteva (11) modified by Gao et al. (12). Briefly, phytic acid is extracted with acids, then transformed to its sodium salt to react with the Wade reagent (Wade: 0.03% FeCl_3_·6H_2_O sulfosalicylic acid 0.3%), and read at 500 nm using a spectrophotometer (Genesis 10 UV, Thermo Electron Corp, Wisconsin, USA).

### Biochemical determinations

A fasting blood sample was collected by venipuncture from each subject. Women were instructed not to eat anything at least 12 h before blood sample was collected early in the morning. Plasma and serum were separated in blood samples by centrifugation at 1800–2000 rpm for 15 min and aliquots were stored at −70°C for later analysis. Blood analysis included hemoglobin, ferritin, and C-reactive protein (CRP). All laboratory analyses were performed in duplicate at the Human Nutrition Laboratory of UAQ.

Hemoglobin and complete blood count were determined using Cell-Dyn (Cell-Dyn 1400, Abbott, EUA) and the corresponding standards. Ferritin was determined with a commercial kit (Ferritin, Biosystem, Barcelona, Spain) and CRP was quantified in serum using a commercial high sensitivity kit (Spinreact, Sant Esteve de Bas, Spain). Both ferritin and CRP were analyzed by spectrophotometry (Genesis 20, Thermo Electron Corp, Wisconsin, USA).

### Iron isotope preparation

Iron isotopes of ^57^Fe (95.6%) and ^58^Fe enriched (93.13%) were purchased in the elemental form (Trace Sciences International Corp, Ontario, Canada) and prepared at the Human Nutrition Laboratory at UAQ. Both forms of metal iron were converted to ferrous sulfate. The ^58^Fe isotope was dissolved at room temperature in 5 mL of 7 mol/L HNO_3_ and 21 mL of 0.5 mol/L H_2_SO_4_ and dried uncovered at 120°C in a muffle furnace for 1 h followed by drying at 230°C for 30 min and 500°C for 30 min. The whitish powder was then reconstituted in 40.5 mL of 0.2 mol/L H_2_SO_4_. The ferrous sulfate solution obtained was brought to a final volume of 335 mL and 0.5 mg/mL concentration, which was checked by atomic absorption spectrometry (Perkin Elmer, Norwalk CT). Finally, ferrous sulfate solution obtained was filtered through with Millex FH-13 filter (Millipore, New Bedford, MA). The ^57^Fe isotope was dissolved in 13 mL of 7 mol/L HNO_3_ and 54 mL of 0.5 mol/L H_2_SO_4_ and dried uncovered at 120°C in a muffle furnace for 1 h followed by drying at 230°C for 30 min and 500°C for 30 min. The whitish powder was then reconstituted in 104 mL of 0.2 mol/L H_2_SO_4_. The ferrous sulfate solution obtained was brought to a final volume of 286 mL and 1.5 mg/mL concentration, which was checked by atomic absorption spectrometry (Perkin Elmer, Norwalk CT). Finally, ferrous sulfate solution obtained was filtered through with Millex FH-13 filter (Millipore, New Bedford, MA). All acids and water used for the preparation of the isotope solutions were Ultrapure Reagent (Merck, Darmstadt, Germany). Final solutions were stored with protection against light at 4°C. ^57^Fe was enriched to 95.7% ^57^Fe and ^58^Fe was enriched to 93% ^58^Fe.

### Iron absorption analysis

To calculate iron absorption, iron isotope ratios were measured at the Children's Nutrition Research Center Department of Pediatrics, Baylor College of Medicine, Houston, TX, USA, using high-resolution double-focusing inductively coupled plasma mass spectrometry (ICP-MS), as previously described (8, 13–15). Red blood cell (RBC) iron incorporation of ^57^Fe and ^58^Fe was measured 14 days after isotope administration and calculated using a mean blood volume of 65 mL/kg, measured hemoglobin concentration, and isotope enrichment. Estimated iron absorption was calculated assuming that 90% of absorbed iron was incorporated into RBC (16). The fraction of iron absorbed from the test meal was calculated relative to the reference dose considering a fixed reference value absorption of 40% to account for differences in iron status of the participants. Total amount of iron absorption was then calculated by multiplying the fractional absorption by the iron content of the meal.

### Statistical analysis

Outcome variables to analyze were the percentage of absorbed iron and the amount of absorbed iron, which was calculated considering the individual intake of iron. General characteristics and baseline ferritin and hemoglobin concentrations of subjects were compared between experimental groups with Student's t-test. To compare the percentage of absorption and amount absorbed between groups, a Student's t-test for independent groups was performed. An additional ANOVA test to compare absorption between groups was performed adjusting for ferritin concentration to evaluate the effect of baseline ferritin concentration on iron absorption. To evaluate the relationship between iron absorption and the percent absorption of the reference dose with baseline ferritin concentration, Pearson correlations were performed. Significant differences were considered at *p<*0.05. All analyses were done using IBM, SPSS, v19.

## Results

**Fig. 1:**
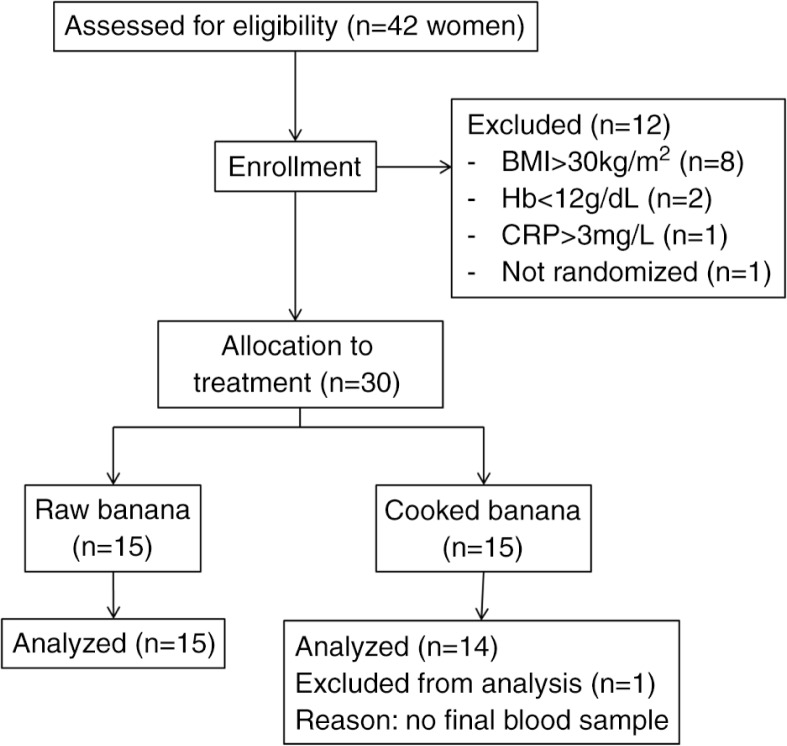
Study flowchart. BMI, body mass index; Hb, hemoglobin; and CRP, C-reactive protein.

Weight and height were measured to calculate BMI. Of the 42 women that were screened, 19.0% were obese (BMI>30 kg/m^2^) and were not included in the study (Fig. 1). A fasting blood sample was taken from the women with adequate BMI for the measurement of ferritin, hemoglobin, and CRP concentration. Of these women, 4.8% had anemia (hemoglobin<11 g/dL) and 2.4% had elevated CRP concentration (CRP>3 mg/L) and hence were not included in the study. None of the subjects had low ferritin concentrations and were not considered with iron deficiency. A total of 32 women met the inclusion criteria, from which 30 were randomly chosen and included in the study. Results of one subject in the cooked banana group were not included in the statistical analyses because the final blood sample was not taken.

A description of the total iron content and phytate content of the bananas used in the study is given in Table 1. Green bananas used in the study had an iron and phytate content of 0.468±0.035 mg and 0.36±0.007 mg per 100 g of banana (dry basis), respectively. Cooked bananas had significantly higher iron content than raw bananas and phytate content was similar in all the banana samples. Total iron intake from bananas of each group was 1.6 mg of iron/480 g of raw banana and 2.6 mg or iron/500 g of cooked banana.

**Table 1:** Total iron and phytate content and phytate to iron molar ratio in raw and cooked bananas used in the absorption studies^a^

Food	Raw banana	Cooked banana	*P*
Iron (mg/100 g banana)	0.33±0.04	0.57±0.02	0.05
Phytate (mg/100 g banana)	0.23±0.02	0.24±0.01	0.482
Phytate to iron molar ratio	0.05	0.03	

a Values are means±SD or molar ratios. Iron and phytate content are indicated in dry basis.

At baseline, BMI and hemoglobin concentration did not differ among groups (Table 2). Ferritin concentration was significantly higher in the group that received the cooked banana compared to the group that received the raw banana with added iron (*p<*0.05).

**Table 2:** General characteristics of participants at baseline^a^

Characteristics	Cooked banana	Raw banana	*P*
*N*	14	15	
Age, years	35.6±5.5	34.4±7.8	0.539
Weight, kg	61.9±7.6	63.8±5.7	0.347
Height, cm	1.5±0.1	1.5±0.03	0.942
Body mass index, kg/m^2^	26.1±3.0	26.8±2.4	0.342
Ferritin, µg/L	91.6±20.9	70.2±12.9	0.006
Hemoglobin, g/dL	14.0±0.8	14.4±1.2	0.311

a Values are mean±SD

**Fig. 2:**
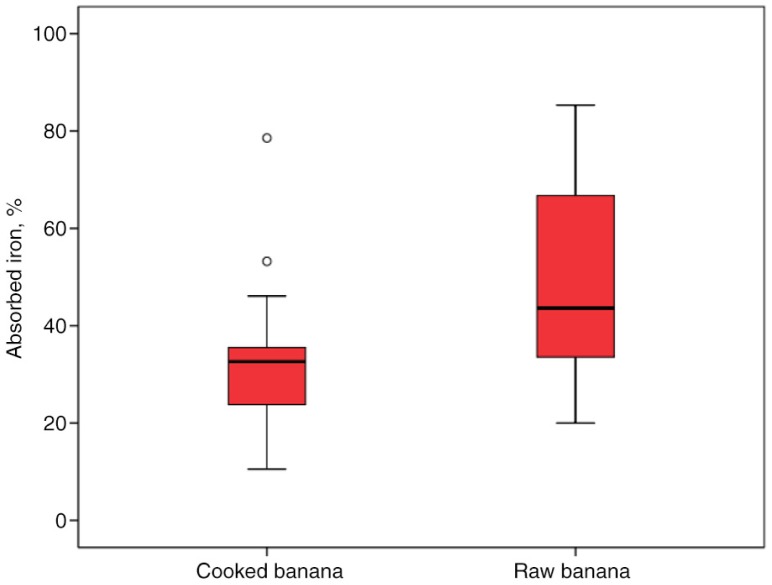
Percent iron absorption in women after intake of cooked and raw bananas for breakfast. ○ represent outliers.

**Fig. 3:**
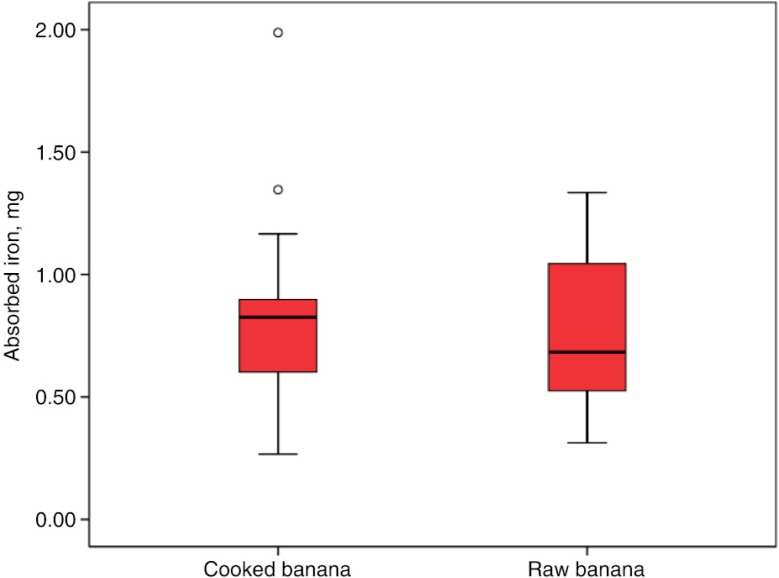
Total amount of absorbed iron in women after intake of cooked and raw bananas for breakfast. ○ represent outliers.

Percent iron absorption was significantly higher in the group consuming raw (49.3±21.4%) compared to the group consuming uncooked bananas (33.9±31.4%; *p=*0.035) (Fig. 2). Total amount of iron absorbed in the cooked bananas and raw bananas was similar (0.86±0.41 mg vs. 0.77±0.33 mg, respectively; *p=*0.525) (Fig. 3).

Reference dose absorption of the participants in the cooked bananas was 42.7±17.2% and in the raw bananas absorption was 31.9±24.1%. No significant differences were observed in the percent absorption of the reference dose between groups.

Since there was a difference in ferritin concentrations between groups, an ANOVA was done adjusting for baseline ferritin concentrations to determine if ferritin had influence in iron absorption; the results indicated no significant effect of ferritin and the estimated mean values were similar that the unadjusted analyses. No correlation was found between iron absorption and ferritin concentration (Pearson correlation coefficient=0.105). Also, no correlation was found between the percent absorption of the reference dose (^57^Fe) and ferritin concentration (Pearson correlation coefficient=−0.17).

## Discussion

Using bananas as a vehicle for iron fortificants could be a potentially effective strategy to increase iron intake in populations with high intake of bananas and high prevalence of iron deficiency and anemia. In the present study, total amount of iron absorbed from raw and cooked bananas was similar.

In the population studied, a difference in iron status was observed between groups. However, the adjusted analysis for ferritin concentration shows that the differences between groups did not affect iron absorption. In addition, iron absorption from the bananas and the reference dose were unrelated to ferritin levels. This is probably due to the fact that none of the subjects had iron deficiency. Thus, differences in iron status are not affecting iron absorption in any of the study groups. Also, in the present study iron absorption was not affected by phytate content. The phytate:iron molar ratio was similar between groups and well below the ratio known to affect iron absorption (phytate:iron>1) (17).

The composition of bananas and its changes due to ripening and cooking may have an important influence on iron absorption. Bananas are a good source of starches and its content changes depending on the degree of ripening and cooking. Ripening and softening of raw bananas is mainly due to an efficient degradation of insoluble starches and accumulation of more soluble and smaller carbohydrates (18, 19). Studies have shown that the accumulation of soluble carbohydrates is significant after approximately 4 days after harvest (DAH) (20). Shiga et al. (19) showed that bananas with an initial amount of starch of 220 g/kg resulted in an increase of 180 g/kg of more simple carbohydrates at 17 DAH. Animal models have shown that iron is bound to some insoluble starches and limit its absorption in the intestinal mucosa (21). When starch is degraded in high amounts of soluble carbohydrates due to ripening, iron solubility increases. Our results show that iron absorption from raw bananas is high probably due to this significant increase of soluble carbohydrates that increase iron solubility. Approximately 50% of the iron that was consumed in the raw bananas was absorbed. Thus, even though raw bananas have a low iron content, absorption is high.

Raw banana starch is resistant to degradation in the small bowel during digestion, and may limit iron absorption. Green bananas have a high content of resistant starch (4.7 g/one medium banana) (22). Green bananas account for 80% of banana intake in Uganda (F.F. De Moura, Harvest Plus, unpublished results). Cooking the bananas modifies its matrix, allowing for an iron absorption of approximately 30%. Cooking has been shown to increase solubility of starch at high temperatures (70–90°C) (23, 24), but to a lesser extent than ripening (18). As mentioned before, when the amount of soluble carbohydrates increase, solubility of iron improves and the amount of iron available for absorption increases. Cooking also increases the amount of resistant starch in bananas (23, 24). Few studies have looked at the effect of resistant starch on iron absorption and results are not consistent. The effect of starch on iron absorption was first studied in the 1960s, where iron absorption in rats was not affected by adding starch to the meals (25). Orzel et al. observed similar results where iron absorption was not affected when adding modified resistant starch to the diet of rats (26). In animal studies, an increase in iron absorption has been observed when fed a high resistant starch diet compared to diets with high soluble starches (27, 28). Morais et al., for example, found that iron absorption doubled when adding 16.4% of resistant starch to the diet of piglets (28). In addition, it has been observed that rats consuming resistant starch significantly decreased the inhibitory effect of phytic acid on iron absorption and iron balance was restored to the control values (27). One possible mechanism that explains why resistant starch improves iron absorption has to do with the solubility of iron. It has been suggested that resistant starch decreases the pH of the intestine, which in turn improves the solubility of iron, and thus, increases its absorption (27). In contrast, iron absorption was reduced in a rat model when starches were added to their diets as a source of carbohydrates and cooking reduced more iron absorption compared to uncooked starches (29). In humans, using the ileostomy model, resistant starch 2 (RS2) from raw green banana flour did not interfere with the absorption of iron in the small intestine, and was not different than cooked green bananas (30). It has also been suggested that iron status may influence the effect resistant starch has on iron absorption. Iron-deficient rats absorbed less iron when fed starch compared with iron-deficient rats fed sucrose (31). Absorption was not affected by starch in rats with adequate iron status. More studies are needed to determine the effect of resistant starch on iron absorption from different food sources including bananas.

One major limitation of the study is the amount of iron consumed per group. The group consuming cooked bananas consumed approximately 1 mg more of iron during the study than the group consuming the raw bananas. However, at the end of the study both groups had similar total iron absorption since absorption of the raw bananas was significantly higher.

## Conclusions

To sum up, total iron absorption is similar in both cooked and raw bananas. The banana matrix does not affect iron absorption and is a potential effective target for genetic modification for iron biofortification. Further research is necessary to describe the mechanism by which cooking modifies the banana matrix and how this affects iron absorption.
